# Body Image Dissatisfaction Is Increased in Male and Overweight/Obese Adolescents in Botswana

**DOI:** 10.1155/2013/763624

**Published:** 2013-03-24

**Authors:** L. Malete, K. Motlhoiwa, S. Shaibu, B. H. Wrotniak, S. D. Maruapula, J. Jackson, C. W. Compher

**Affiliations:** ^1^University of Botswana, Gaborone, Botswana; ^2^Johns Hopkins Bloomberg School of Public Health, Baltimore, Maryland 21205, Botswana; ^3^Division of Gastroenterology, Hepatology, and Nutrition, The Children's Hospital of Philadelphia, Philadelphia, PA 19104, USA; ^4^D'Youville College, Buffalo, NY 14201, USA; ^5^School of Nursing, University of Pennsylvania, Philadelphia, PA 19104, USA

## Abstract

*Introduction*. The purpose of this study was to examine linkages between obesity, physical activity, and body image dissatisfaction, with consideration of socioeconomic status (SES) and urbanization in adolescents in Botswana. *Materials and Methods*. A nationally representative, cross-sectional survey in 707 secondary school students included measured height and weight to determine overweight (OW) or obesity (OB) using World Health Organization standards; physical activity (PA) using the International Physical Activity Questionnaire; and body image satisfaction using the Body Ideals Questionnaire. SES was described by private school versus public school attendance. *Results and Discussion*. OW/OB students felt farther from ideal and greater dissatisfaction with their weight and body proportions than optimal weight students. Boys felt greater difference from ideal and more dissatisfaction with muscle tone, chest size, and strength than girls. Lower SES students and those from rural villages had more minutes of PA than higher SES or urban students. In this rapidly developing African country, these trends reflect the nutrition transition and offer opportunity to motivate OW/OB students and boys for PA as a health promotion obesity prevention behavior. *Conclusions*. As urbanization and improved SES are desirable and likely to continue, the public health system will be challenged to prevent obesity while preserving a healthy body image.

## 1. Introduction 

Developing countries undergoing economic transition are grappling with the phenomenon of nutrition transition, characterized by a shift from under-nutrition to overweight (OW) and obesity (OB), or their coexistence [1]. Given that OB is a risk factor for metabolic syndrome, diabetes, hypertension, and other cardiovascular diseases, its growing prevalence in developing countries leads to more complex problems for their health systems that remain overburdened by infectious disease.

OW and OB are associated with body-image dissatisfaction and low self-esteem [2]. In adolescents from 24 countries and regions across Europe and North America, 43%–51% of those surveyed were dissatisfied with their body weight [2]. Although this pattern was thought to occur predominantly in western culture, it is increasingly affecting developing countries [3, 4]. Dieting and physical activity behaviors for weight management, and a preference for thinness, are increasingly prominent across the globe, especially among women and adolescent girls. Unfortunately, there has been little research on body image dissatisfaction in African populations, though diet and exercise behaviors found in western populations may also exist there. 

Cash and Szymanski [5] defined body image as a “multifaceted psychological construct that includes subjective attitudinal and perceptual experiences about one's body, particularly its appearance.” Body image concerns an individual's perceptions, thoughts, and feelings about one's body across dimensions of body size, attractiveness, and body. The construct of body image is based on the self-discrepancy theory [6], a conceptualization that seeks to explain negative emotions experienced by individuals who hold two conflicting beliefs about themselves. One belief is based on how one actually sees his or her self, considered to be the “actual self,” and the other is based on attributes as judged by others considered to be the “ideal self.” When the two selves do not match, there is a discrepancy with the resultant effect of negative emotions or dissatisfaction. The self-discrepancy theory has been widely used to conceptualize body image dissatisfaction, to develop assessment instruments, and to demonstrate various facets of the construct [5, 7]. 

Body image may be linked with socioeconomic status (SES) and urbanization. In a cross-cultural study comparing body image perception among women from 26 countries in 10 regions across the globe, ideal body weight was reported to be slimmer in contexts of high compared to low SES, in more westernized societies and among individuals exposed to idealized body image by the media [4]. Among Malaysian and South African participants, rural participants in both countries reported significantly higher actual and ideal body ratings, as well as lower body dissatisfaction. In fact, thinness may be negatively perceived as a sign of ill health, especially among individuals from low SES, while a heavier body is culturally perceived as a sign of health, beauty, prestige, and prosperity [4, 8, 9]. However, in developed countries, body dissatisfaction has been suggested as a protection against excessive weight gain and binge eating behaviors [10, 11]. These conflicting findings suggest the need for more investigations into body image dissatisfaction and how it relates to OW/OB and other factors such as physical activity (PA) and general self-esteem. 

Urbanization has been associated with reduced PA [12]. Higher body dissatisfaction and pressure to lose weight have been linked to a lack of motivation for PA among adolescents with higher BMI [13]. Girls experiencing higher body dissatisfaction and criticism about their weight are less likely to engage in vigorous PA, compared to those who faced criticism without body dissatisfaction [14]. These findings indicate the possible impact of higher body dissatisfaction and/or pressure to lose weight on the likelihood to engage in PA. Given that physical inactivity is a risk factor for OW/OB, attention has to be paid to understanding the possible reciprocal relationship between body image dissatisfaction, weight status, and PA. Such an understanding, especially with adolescents, will be critical to the developing of appropriate, multifaceted, and long-term obesity intervention strategies. 

Like many developing countries, Botswana is undergoing the nutrition transition with a rapid increase in the prevalence of OW/OB in adolescents. Our group has demonstrated adolescent OW and OB prevalence of 12% and 5%, respectively, with much higher levels in urban areas (17% and 9%, resp.) compared with rural areas (5% and 0%, resp.) [15], and that school personnel are very concerned about the issue in students [16]. We have also shown that adolescents in Botswana are influenced by teachers, coaches, and parents to participate in PA, especially competitive sports [17]. However, interventions to prevent obesity among children and adolescents in Botswana are lacking. Furthermore, the prevalence of body dissatisfaction and how it may vary by gender, PA, SES, or urbanization has not been reported. Thus, the purpose of this study was to examine whether OB or PA predict body image dissatisfaction in adolescent students in Botswana and to determine whether age, gender, SES, and level of urbanization modify the likelihood of body image dissatisfaction.

## 2. Materials and Methods 

The study protocol was approved by the Institutional Review Board at the University of Pennsylvania and the Ministry of Education in Botswana. All the students approached for participation were given IRB-approved parental consent forms written in English and Setswana to be returned to the school if consent was granted. All participating students also had to give assent.

The study had a two-stage stratified sampling process to select participating schools with the aim to include both urbanization and SES representation. We followed the designation of the Botswana Housing Census in assigning levels of urbanization. In Botswana, localities are largely classified by levels of urban infrastructure and population density (Botswana CSO Housing Census 2001). Cities have the highest levels of urbanization and population density of 80,000–200,000 people. Towns have similar infrastructure to cities, but the population is 15,000–50,000. Urban villages have more traditional infrastructure and similar population to towns. Rural villages maintain the most traditional infrastructure and have populations <5,000 people. 

In developing countries such as Botswana, individual SES varies both by income levels and by the level of infrastructure development within one's area of residence. Public schools do not require payment of tuition and represent a mixture of SES circumstances with a significant representation of lower SES, while private schools require payment of tuition fees that are generally out of reach for lower SES families. However, in rural villages, private schools are not typically available. The physical infrastructure that provides electricity and running water directly into family residences may be more limited in rural villages as well as in impoverished sections of cities, towns, and urban villages than in wealthier areas. 

The first stage of the sampling process involved random selection of districts on the basis of the Botswana educational statistics at the time of the survey [18], and the second stage involved random selection of schools within these districts. From the 274 total secondary schools in Botswana, 17 secondary schools were randomly selected to represent urbanization: seven from cities, four from towns, three from urban, and three from rural villages. Of the 17 schools sampled, 12 were public and 5 were private schools, to represent lower and higher SES. Within each school selected, a convenience sample of approximately 50 students was enrolled. 

Data were collected by two research assistants who were trained on questionnaire administration and protocol-based collection of anthropometric data by expert researchers at the University of Botswana. 

Anthropometric interassessor reliability was assessed by measuring 20 randomly sampled volunteers of the target population as part of pilot testing from a school that did not participate in the final data collection and comparing the results to those of the expert anthropometrists. Of the variables reported in this paper, certification was granted when measurements were within ±0.3 cm for height. Anthropometric equipment was checked and calibrated before every assessment session. All data collection sessions at schools were supervised by faculty researchers from the University of Botswana to ensure continuous quality and reproducibility. 

All data collection sessions were conducted in a semiprivate section of the classroom or school cafeteria to ensure privacy regarding physiological measures. Body weight was measured to the nearest 0.1 kg in light clothing on a digital scale (Tanita, Tokyo, Japan). Height was measured without shoes using a stadiometer (Seca, Hamburg, Germany). 

BMI was calculated as weight (kg)/height (m^2^) and evaluated against World Health Organization (WHO) norms for age and gender using AnthroPlus software (Geneva, Switzerland: World Health Organization, 2009) to determine adolescent weight status: underweight, desirable weight, overweight, and obese. We confirmed the OW and OB classifications using the International Obesity Taskforce cut-offs [19].

Participants completed a demographic survey that included gender, age, and SES variables. SES was evaluated by attendance at a tuition-free public (low SES) or tuition-requiring private (high SES) school and by self-reported responses of household assets (television, refrigerator, electricity, water, and toilet). We coded 3–5 assets as high SES and 0–2 assets as low SES. 

Body image satisfaction was assessed using the Body-Image Ideals Questionnaire (BIQ) with permission of the author [5]. The BIQ requires respondents to evaluate how different their ideal body is from their perceived actual body, and how important it is for them to attain the perceived ideal body. The questionnaire uses a 4-point Likert scale to assess each of 11 physical attributes (facial features, hair texture and thickness, skin complexion, height, muscle tone and definition, body proportion, weight, chest size, physical strength, physical coordination, and overall appearance). The range for composite BIQ score is from −3 (very important congruence across all physical attributes) to +9 (very important and maximum discrepancies across all physical attributes). Thus, higher BIQ scores indicate higher body image dissatisfaction. 

Physical activity was measured using the International Physical Activity Questionnaire—Short Form (IPAQ), which characterizes PA based on days and hours spent per day on activities classified as “walking,” “moderate activity,” and “vigorous activity” [20]. Based on IPAQ scoring guidelines, data for walking, moderate, and vigorous time variables exceeding 3 hours per day were truncated to reflect maximal daily activity levels of 3 hours per day for each PA category. Sedentary behavior was represented by the total minutes per week spent sitting from the IPAQ. 

The sample size was calculated using the population survey approach in STATCALC (EpiInfo, 2002, CDC, Atlanta, GA, USA). Because the actual prevalence of OW and OB was unknown, the prevalence was estimated at the high rate of 50%. To have 90% power to detect a significant difference at *P* < 0.05, the calculated sample size was 661. Taking into account a nonresponse rate of 10% observed in our prior research there, the target recruitment was 728 adolescents. In total, 754 students enrolled in the study. 

Measures of central tendency are reported as mean (SD) or number (%). Differences in minutes of PA and BIQ difference from ideal and dissatisfaction were compared across gender, SES, urbanization, and BMI groups using unpaired *t*-tests or ANOVA. To test whether body image dissatisfaction varies with physical activity levels or BMI group, separate linear regression models predicting composite BIQ score were used. Next, the models were adjusted for age, gender, SES, and level of urbanization. For all comparative analyses and statistical models, because urban villages and towns represent similar levels of economic development and because there were no statistical differences between, these groups in BIQ scores, these groups were combined. Likewise, because of low rates of OW and OB in rural areas, these groups were combined to increase sample size for statistical analyses. Data were analyzed using STATA statistical software version 11 (StataCorp LP, College Station, TX, USA, 2000) and SPSS version 19 (IBM Statistics, Chicago, IL, USA). Because of the multiple comparisons, statistical significance for all analyses was set at *P* < 0.01.

## 3. Results

A total of 857 students were approached to participate, of whom 754 returned parental consent forms and gave student assent, a response rate of 88%. A summary of demographic information is provided in Table 1. The mean age of students was 14.9 years, and 61% were girls. The low SES variables included 30% with low household assets and 69% attending public schools, while 70% had high assets and 31% attended private schools. The representation of students within the urbanization groups was rural villages (24%), urban villages (24%), towns (20%), and cities (32%). For BMI groups, 5% were underweight, 78% were desirable weight, 12% were overweight, and were 5% obese. The mean total physical activity reported was 695 minutes/week (1.7 hours/day), by contrast to 2612 minutes/week (6.2 hours/day) spent sitting. The duration of PA was examined across gender, SES, urbanization, and BMI groups (Table 2). There were no significant differences in any level of PA in boys versus girls or in the BMI groups (data not shown). Public school students (lower SES) had significantly more minutes of sitting, vigorous PA, and total PA than students at private schools (all *P* < 0.01). Students living in rural villages reported more minutes/week of sitting than students from cities. While minutes of walking were not significantly different by urbanization group, students from rural villages had more minutes of moderate PA, vigorous PA, and total PA than students from towns/urban villages or cities. 

The composite BIQ score was 1.53 (SD 2.06) (Table 1). The BIQ variables did not differ significantly by SES group (data not shown). However, significant differences in students' perception of their own features relative to ideal and of how dissatisfied they were with these features are reported by gender, urbanization group, and BMI group in Table 3. Boys reported greater difference from and dissatisfaction with their ideal muscle tone, chest size, and strength, relative to girls (*P* < 0.01). Students from towns/urban villages indicated greater difference from their ideal body proportions and overall appearance (both *P* < 0.01) than adolescents from cities; however, their level of dissatisfaction with these two factors did not reach statistical significance. OW/OB students reported greater difference from (*P* < 0.01) and dissatisfaction with (*P* < 0.001) ideal body weight and proportions than optimal weight students. In regression analyses, none of the potential predictor variables (total PA, BMI group, age, gender, school type, and urbanization), separately or in combination, predicted composite BIQ score (data not shown). 

## 4. Discussion

Our purpose was to examine whether OB or PA predict body image dissatisfaction in adolescent students in Botswana and to determine whether age, gender, SES, and level of urbanization modify the likelihood of body image dissatisfaction. We found boys were more dissatisfied with their muscle tone, strength, and general appearance than girls. Students from higher SES and more urbanized areas had less vigorous and total PA, and OW/OB students perceived their proportions and body weight as farther from the ideal and expressed greater dissatisfaction with these attributes than optimal weight students. Understanding the relationship between OW and OB, PA and body image satisfaction is important for intervention designs because perception about one's self-image affects motivation for physical activity [17, 21]. 

These findings describe significant linkages between urbanization and reduced PA that are most likely associated with increased vehicular transport and/or reduced personal safety of students in an urban environment [22]. The lower PA reported by students from higher SES private school backgrounds suggests that increasing family wealth may play a role in access to motorized transportation or required indoor activity as parents are working outside the home. This is particularly ironic since PA is compulsory in private schools but only optional in public lower SES schools in Botswana. Since data from this same sample have also described increased OW/OB among urbanized and higher SES children [15] and increased intake of snack foods and fewer traditional Botswana meals in cities [23], this series of findings is consistent with the nutrition transition and bodes a public health challenge as urbanization is likely to continue. 

Our findings are in concert with some other studies. In adolescent girls in Britain, a strong association between body dissatisfaction and body fatness was reported, but no association between body dissatisfaction and PA [21]. In South Asian children, perceived body size was positively related to weight status, and OW/OB youths were more likely to select a thinner ideal body size than healthy weight children [24]. A study of motivation for physical activity in the UK found that adolescent boys with higher body satisfaction were more likely to maintain leisure-time PA, whereas dropping out of physical exercise and a reduced likelihood of taking up exercise were predicted by higher BMI and pressure to lose weight [13]. The cross-sectional nature of our study does not enable us to make this comparison in terms of change in or maintenance of PA. Any disconnect between PA and body image satisfaction may impact the effectiveness of PA interventions due to lack of motivation, as it has been argued that adolescent body image perceptions have an effect on their motivation for physical activity [17, 21]. 

A recent study in Taiwan found that adolescent girls were more likely to be dissatisfied with their body and their physical appearance than boys, and that higher BMI was associated with greater body dissatisfaction [3]. In this survey in Botswana, higher BMI was associated with more dissatisfaction with body proportions and weight, but girls were not more dissatisfied than boys, even though they had a greater prevalence of OW [15]. Another study found that girls experiencing higher body dissatisfaction and criticism about their weight were less likely to engage in vigorous PA, compared to those who faced criticism without body dissatisfaction [14]. We did not ask about criticism experienced by girls in this study; however, their PA was not different from boys, nor was the composite BIQ score.

Our group has previously shown that the prevalence of obesity differs substantially between urban and rural areas, with urban areas having higher OW/OB prevalence than rural areas [15]. However, levels of urbanization were not significantly linked with body dissatisfaction scores in this study, though students from towns/urban villages reported greater discrepancy from the ideal body proportions and overall appearance. This may suggest an early awareness of discrepancy that risks increasing to outright dissatisfaction if cultural influences are increased in a negative direction.

OW/OB adolescents were more dissatisfied with their body proportions and weight. This suggests that interventions targeting weight loss for OB adolescents may benefit from incorporating body image training into program design as a way to motivate adolescents to commit to the interventions. It would, however, be important to measure and account for the relationship between perceived weight status and actual weight status so that such interventions do not lead to eating disorders by normal weight adolescents wanting to “attain the ideal.” This is particularly important as it has been demonstrated that perceived weight status was a better predictor of weight loss intent than actual body fatness in a multiethnic sample of adolescent girls [21]. A study of Brazilian adolescents found that girls tended to overestimate their weight, while boys were more likely to underestimate theirs [25]. Little is known about the influence of cultural pressures and norms on the development of body satisfaction or dissatisfaction in Botswana thus, it is imperative to consider both actual and perceived body weights in interventions with OW/OB students. 

These findings should be viewed in light of the study's strengths and limitations. The strengths of this study include its population-based design with surveys for adolescents across regions and SES contexts collected in the school setting. At the time of this, study Botswana census data showed that 94% of 12- to 18-year-olds attend school [18], and that the percentage of the population living below the poverty line had decreased to about 30% in 2002-2003 similar to our SES data [26] thus, our school-based survey is likely to provide a good representation of the national obesity prevalence in this age group. Height and weight were obtained by measurement, using well-trained research assistants and a rigorous protocol. Unfortunately, body weight data were lost on 38 students at one school due to a malfunctioning scale, requiring that BMI be excluded to, though all other student information were included. The major limitations of the study are related to the self-reported measures of body image satisfaction, physical activity, and socioeconomic status. Objective measurement of physical activity, using accelerometers, would provide stronger data but was beyond the scope of this project. Since adolescents are unlikely to be knowledgeable about family income or parental education levels, we needed to limit SES questions to items that they could easily describe. 

## 5. Conclusion

Our study has shown that OW and OB among adolescent students in Botswana is associated with body image dissatisfaction, particularly of body proportions and weight, while PA is not. The discrepancy from ideal and dissatisfaction that boys have with muscle tone, chest size, and strength might be used as a motivation for increased physical training and PA. These associations should be used to inform targeted obesity prevention interventions as well as programs for adolescent psychosocial development in Botswana.

## Figures and Tables

**Table 1:** Demographic information.

Variable	Mean (SD) or *N* (%)
Age (years)	14.91 (1.32)
Female/male	464 (61.4%)/292 (38.6%)
Socioeconomic status	
Public/private school	523 (68.8%)/237 (31.2%)
Household assets (low/high)	225 (30.4%)/515 (69.6%)
Urbanization of residence	
Rural village	184 (24.2%)
Town	148 (19.8%)
Urban village	182 (23.9%)
City	246 (32.4%)
BMI group	
Underweight	35 (5%)
Desirable weight	554 (78.4%)
Overweight	82 (11.6%)
Obese	36 (5.1%)
Physical activity (minutes/week)	
Sitting	2612.38 (910.87)
Walking	287.94 (297.89)
Moderate PA	188.96 (267.79)
Vigorous PA	219.59 (275.89)
Total PA	695.45 (568.82)
Composite BIQ score	1.53 (2.06)

**Table 2:** Physical activity by socioeconomic status and urbanization.

Physical activity (min/week)	Socioeconomic status		Urbanization		
	Public school	Private school	City	Town/urban village	Rural village
Sitting	2705.1 (863.8)	2446.9 (996.9)**	2325.7 (1044.8)	2772.1 (880.4)	2758.1 (691.9)*** versus city
Walking	297.7 (308.3)	265.7 (274.2)	284.6 (302.6)	292.8 (296.9)	283.6 (298.4)
Moderate PA	207.0 (286.4)	155.9 (222.3)	167.7 (259.4)	164.0 (222.9)	271.4 (336.8)** versus other groups
Vigorous PA	245.3 (293.4)	158.8 (219.4)***	201.9 (268.3)	194.3 (252.9)	288.4 (314.9)** versus other groups

Total PA	746.2 (594.9)	588.1 (499.6)**	661.7 (601.7)	648.9 (484.9)	832.9 (654.1)*** versus other groups

Data as mean (SD) and *P* values by unpaired *t*-test or ANOVA.

**P < 0.01; ***P < 0.001.

**Table 3:** Body Image Questionnaire difference from ideal and dissatisfaction scores.

BIQ factor	Gender		Urbanization			BMI group		
	Male	Female	City	Town/urban village	Rural village	Underweight	Optimal BMI	Overweight/ Obese
Difference from Ideal								

Height	0.9 (1.5)	0.7 (1.4)	0.6 (1.4)	0.8 (1.5)	0.8 (1.4)	1.1 (1.5)	0.8 (1.4)	0.5 (1.4)
Skin complexion	0.5 (1.4)	0.5 (1.4)	0.5 (1.4)	0.6 (1.4)	0.6 (1.5)	0.2 (1.4)	0.6 (1.4)	0.3 (1.3)
Hair texture	0.4 (1.4)	0.7 (1.5)	0.5 (1.5)	0.6 (1.5)	0.5 (1.5)	0.2 (1.4)	0.6 (1.5)	0.4 (0.5)
Facial features	0.4 (1.4)	0.6 (0.5)	0.4 (1.4)	0.5 (1.5)	0.4 (1.5)	0.6 (1.5)	1.5 (4.5)	0.4 (1.5)
Muscle tone	**1.2 (1.4)**	0.8 (1.4)***	0.4 (0.1)	1.5 (0.1)	1.4 (0.1)	1.2 (1.2)	0.8 (1.4)	0.9 (1.5)
Body proportions	0.8 (1.4)	0.9 (1.4)	**0.7 (1.40)**	1.1 (1.4)**	0.8 (1.4)	0.7 (1.5)	**0.8 (1.4)**	1.3 (1.4)**
Weight	0.8 (1.5)	0.9 (1.5)	1.5 (0.1)	1.5 (0.1)	1.5 (0.1)	**1.3 (1.4)**	0.6 (1.5)**	**1.6 (1.4)**
Chest size	**0.9 (1.5)**	0.6 (1.5)**	1.4 (0.1)	1.5 (0.1)	1.5 (0.1)	1.0 (1.4)	0.6 (1.5)	0.8 (1.6)
Strength	**1.1 (1.4)**	0.8 (1.4)**	0.8 (1.4)	1.0 (1.4)	0.9 (1.4)	1.2 (1.4)	0.9 (1.4)	0.8 (1.4)
Coordination	0.6 (1.4)	0.7 (1.4)	0.6 (1.4)	0.7 (1.5)	0.7 (1.4)	0.5 (1.4)	0.6 (1.4)	0.7 (1.3)
Overall appearance	0.9 (0.4)	0.8 (1.5)	**0.8 (1.4)**	1.1 (1.4)**	**0.7 (1.4)**	0.7 (1.5)	0.8 (1.4)	1.0 (1.4)

Dissatisfaction								

Height	1.8 (3.4)	1.4 (3.3)	1.1 (3.3)	1.8 (3.4)	1.7 (3.4)	1.8 (3.4)	1.6 (3.4)	1.1 (3.3)
Skin complexion	0.9 (3.2)	0.9 (3.2)	0.9 (3.3)	1.1 (3.2)	1.0 (3.2)	−0.03 (3.0)	1.2 (3.2)	0.5 (3.1)
Hair texture	0.8 (3.1)	1.3 (3.5)	1.2 (3.4)	1.1 (3.3)	0.9 (3.4)	−0.15 (2.6)	1.1 (3.3)	0.9 (3.4)
Facial features	0.7 (3.3)	1.0 (3.6)	0.8 (3.4)	1.0 (3.5)	0.8 (3.6)	0.6 (3.3)	0.9 (3.4)	0.8 (3.6)
Muscle tone	**2.6 (3.7)**	1.5 (3.3)**	1.5 (3.2)	2.2 (3.6)	1.9 (3.5)	1.6 (3.0)	1.8 (3.4)	1.9 (3.8)
Body proportions	1.7 (3.4)	1.9 (3.4)	1.6 (3.4)	2.2 (3.4)	1.5 (3.4)	1.2 (3.2)	**1.5 (3.2)**	2.7 (3.7)***
Weight	1.6 (3.5)	2.2 (3.8)	1.7 (3.6)	2.3 (3.8)	1.7 (3.5)	2.4 (3.2)	**1.3 (3.4)**	3.6 (3.7)***
Chest size	**1.8 (3.4)**	0.9 (3.2)***	1.1 (3.2)	1.3 (3.4)	1.4 (3.2)	1.7 (3.2)	1.0 (3.2)	1.4 (3.5)
Strength	**2.5 (3.7)**	1.5 (3.2)***	1.8 (3.4)	1.9 (3.4)	1.9 (3.6)	2.5 (3.5)	1.8 (3.3)	1.9 (3.7)
Coordination	1.3 (3.4)	1.3 (3.1)	1.2 (3.1)	0.3 (3.3)	1.3 (3.2)	0.8 (3.3)	1.2 (3.2)	1.3 (3.1)
Overall appearance	2.1 (3.5)	1.9 (3.8)	1.7 (3.6)	2.4 (3.8)	1.6 (3.5)	1.0 (3.2)	1.7 (3.6)	2.4 (3.6)
Composite BIQ score	1.6 (2.1)	1.5 (2.1)	1.4 (1.9)	1.7 (2.1)	1.4 (2.0)	1.2 (1.7)	1.4 (1.9)	1.7 (2.3)

Data as mean (SD); **P < 0.01; and ***P < 0.001.
